# Clinical, epidemiological characteristics and mortality of pregnant and postpartum women associated with COVID-19 in Brazil: cohort study

**DOI:** 10.61622/rbgo/2024rbgo52

**Published:** 2024-06-27

**Authors:** Gustavo Gonçalves dos Santos, Anderson Lima Cordeiro da Silva, Edson Silva do Nascimento, Luis Henrique de Andrade

**Affiliations:** 1 Faculdade de Enfermagem Universidade Santo Amaro São Paulo SP Brazil Faculdade de Enfermagem, Universidade Santo Amaro, São Paulo, SP, Brazil.; 2 Escola de Enfermagem de Ribeirão Preto Universidade de São Paulo Ribeirão Preto SP Brazil Escola de Enfermagem de Ribeirão Preto, Universidade de São Paulo, Ribeirão Preto, SP, Brazil.; 1, 3 Faculdade de Ciências da Saúde Albert Einstein Hospital Israelita Albert Einstein São Paulo SP Brazil Faculdade de Ciências da Saúde Albert Einstein, Hospital Israelita Albert Einstein, São Paulo, SP, Brazil.

**Keywords:** COVID-19, Coronavirus infections, SARS-CoV-2, Pregnancy, Postpartum period, Maternal death, Health information systems

## Abstract

**Objective:**

To analyze the death of Brazilian pregnant and postpartum women due to COVID-19 or unspecific cause.

**Methods:**

This is retrospective, descriptive-exploratory, population-based study carried out with the Sistema de Informação de Vigilância Epidemiológica da Gripe (SIVEP-Gripe) database, with pregnant and postpartum women of reproductive age who died from confirmed COVID-19 between 2020 and 2021. The chosen variables were: age, gestational period, type and number of comorbidities, skin color, using the statistical software R Foundation for Statistical Computing Platform, version 4.0.3 and Statistical Package for Social Science, version 29.0 for analysis.

**Results:**

A total of 19,333 cases of pregnant and postpartum women aged between 10 and 55 years diagnosed with SARS were identified, whether due to confirmed COVID-19 or unspecific causes. Of these, 1,279 died, these cases were classified into two groups according to the cause of death: deaths from COVID-19 (n= 1,026) and deaths from SARS of unspecific cause (n= 253).

**Conclusion:**

The risk of death increased among black and brown women, in the postpartum period and with the presence of comorbidities, mainly diabetes, cardiovascular diseases and obesity. The data presented here draw attention to the number of deaths from SARS, especially among sociodemographic profiles, precarious access to health, such as the black population. In addition, limitations in adequate access to health care are reinforced by even lower rates of ICU admissions among women who died from SARS of an unspecified cause.

## Introduction

COVID-19 is a respiratory disease caused by the Severe Acute Respiratory Syndrome – Coronavirus 2 (SARS-CoV-2) coronavirus, since its emergence in the city of Wuhan, China, in December 2019, the disease has spread rapidly around the world, leading to a global COVID-19 pandemic.^(1,2)^The COVID-19 pandemic has affected many women’s pregnancy, childbirth and postpartum period. Pregnant women are considered a risk group, as they may have an altered immune response, which makes them more susceptible to viral infections.^(3,4)^

In Brazil, between January 18 and February 21, 2020, the Health Surveillance Secretariat of the Ministry of Health (MS) received notification of 154 cases for investigation of possible infection by COVID-19.^(5)^ Months later, on April 3, there were 359 deaths and 9,056 cases, of which 1,146 were new cases of the disease, confirmed in 24 hours, with the State of São Paulo remaining with the highest number of confirmed cases (44.7%). Followed by Rio de Janeiro (11.9%), Ceará (6.9%), Federal District (4.4%) and Minas Gerais (4.4%).^(5,6)^ A summary of the Brazilian situation, carried out on April 8, indicated that the country occupied the 14th position in the number of confirmed cases, the 12th In the number of deaths, the 8th in relation to the lethality rate and the 16th in the death rate due to COVID-19.^(7,8)^ Data released in epidemiological bulletins (EB) show that MS received the first notification of a confirmed case of COVID-19 in Brazil on February 26, 2020 and that, from that date until September 2020, 4,582,240 cases and 136,532 cases had been confirmed. Deaths from COVID-19 in the country, with the highest number of new cases (69,074 cases) and new deaths (1,595 deaths) occurring on July 29, 2020.^(9)^

In the period from April to May 2021, the proportion of pregnant women among the cases of Severe Acute Respiratory Syndrome (SARS) that required hospitalization was maintained and the death rate among them was 8%,^(10)^ a situation that remained forward, between June and August 2021,^(11,12)^ followed by a 7.6% three months later. Thus, by SE 47 of 2021, among the 1,063 deaths of pregnant women due to SARS resulting from COVID-19, 59 (5.5%) had occurred in black women.^(13)^ In December 2021, the EB pointed out that of the total number of SARS cases reported in pregnant women (15,390) with onset of symptoms up to epidemiological week (EW) 48, 1,114 (7.4%) evolved to death and of the total number of deaths from SARS, 93.3% (1,067) were confirmed for COVID-19, with brown color being more frequent among deaths from SARS due to COVID-19 (46.1%), followed by white (38.3%).^(14)^

In the study by Mullins et al.,^(15)^ case reports or case series were included, totaling 32 pregnant women with COVID-19, one case of a twin pregnancy. There were 27 deliveries by cesarean section and two vaginal deliveries, and in three cases the pregnancy was ongoing. Seven (22%) pregnant women remained asymptomatic and two (6%) were admitted to the Intensive Care Unit (ICU), one of which remained on extracorporeal membrane oxygenation. Most pregnant women showed mild symptoms of the disease, with fever, runny nose, nasal congestion and dyspnea, with no evidence of vertical transmission. No maternal deaths have been reported to date. There were 15 (47%) premature births, there was one stillbirth and one neonatal death. They concluded to SARS and COVID-19 appeared less lethal, however they reported that the results should be viewed with caution given the limited number of reported cases. They recommended that the type of delivery be determined by obstetric indication.^(15)^

In a systematic review, it was shown that pregnant women have a faster evolution to moderate and severe conditions,^(16)^ from 1 to 5% need ventilatory support and/or care in the Intensive Care Unit (ICU), with a higher risk of maternal complications being verified, mainly in the last two trimesters of pregnancy and in the puerperium.^(17)^ In developing countries, the reasons for maternal death, in this pandemic period, have increased,^(18)^ including in Brazil, where epidemiological data point to an increase in cases and maternal death from SARS.

Knowing that pregnancy is a period with several physiological changes, and pregnant and puerperal women, during infections caused by the SARS-CoV, influenza and MERS-CoV viruses, which occurred in 2002, 2009 and 2012, presented various complications such as: fever, cough, dyspnea and the need for an ICU.^(19)^ Recognizing the high risk of morbidity and mortality, most symptoms are mild, such as fever and dry cough, however, in women in the second half of pregnancy, other symptoms may appear with less intensity, such as fatigue, dyspnea, diarrhea, congestion nose and runny nose, some of these women may still have more serious complications, such as SARS.^(20)^

According to a Chinese review study, it described the clinical picture and points out that pneumonia caused by the new coronavirus is a highly contagious disease and, as such, rapidly spreading; that the specific mechanism of action of the virus remains unknown and that specific drugs for treatment had not been developed, as well as vaccines.^(21)^ The scientific literature points out that pregnant women with SARS-CoV-2 infection, in which they progress to a severe condition associated with comorbidity, have an increased probability of having an emergency cesarean delivery or premature labor, which increases the risk of maternal morbidity and mortality and neonatal.^(22)^

In view of the above general panorama, the present study focuses on the effects of COVID 19 on a specific group of pregnant women: those who are black or brown, and whether it stems from the possible identification of a greater risk among these women, who in general experience a condition of social inequality and difficult access to health services. Data released by the United Nations (UN) show that maternal mortality in black women due to COVID-19 was almost twice as high as that observed in white women^(23,24)^ highlighting that the consequences of the COVID-19 pandemic in a structured society due to racism, they penalize vulnerable groups,^(25)^ exposing black women, as a result of their social, political, economic and cultural insertion.^(26)^

It is important to compare the results of Brazilian studies with international studies, because after the spread of the pandemic, the highest number of pregnant women in the world was reached, American studies such as the one by the Centers for Diseases Control and Prevention (CDC^(27)^ the study Swedish^(28)^ and studies in Belgium and France^(29)^ began to demonstrate an increased risk of complications and ICU admission, in addition to a greater need for mechanical ventilation in pregnant women, but not of maternal death. Maternal deaths seem to be more frequent in low and middle-income countries and would be due to serious failures in the health system combined with the social determinants of the health-disease process. This has also been seen in other Latin American countries, especially Mexico, which maintains an efficient notification system.^(30)^

Finally, to define the guiding question, the PCC strategy was used, an acronym for Population, Context and Concept in which: “P” – Brazilian pregnant and puerperal women; “C” – COVID-19 pandemic; “C” - pregnant and puerperal women confirmed for COVID-19 or for non-specific causes with severity of disease progression. In this sense, the objective was to analyze, through the secondary database of the SIVEP-Gripe, the death records of Brazilian pregnant and puerperal women COVID-19 or due to nonspecific causes, and to compare with the characteristics sociodemographic and clinical characteristics of both groups of patients.

## Methods

This is a retrospective, descriptive-exploratory, population-based study and thus explores a problem again, providing information for more precise investigations and establishing the bases for future studies, with the purpose of knowing the contributions scientific studies that were carried out on a given subject, with a quantitative approach and aimed at portraying the behavior and development of scientific production in a given area of knowledge.

Conducted in Brazil, a retrospective cohort analysis of data obtained from the Sistema de Informação de Vigilância Epidemiológica da Gripe (SIVEP-Gripe) secondary database was performed, which groups records of COVID-19, which feeds mandatory notification for flu-like illnesses, which are characterized by at least two signs and symptoms: fever, even if reported, chills, sore throat, headache, cough, runny nose, and smell or taste disturbances, in addition to dyspnea, respiratory distress, oxygen saturation (O2) less than 95% in ambient air and death records.

The study included pregnant and puerperal women aged between 10 and 55 who died from confirmed COVID-19. The blank SARS etiology field was not included in the analysis because it was not possible to determine if they met the unspecified definition or if it was a record quality issue. SARS cases confirmed as Influenza, another virus or etiologic agent were also excluded.

Cases notified between 2020 and 2021 that met the definition of SARS by SIVEP-Gripe were selected, knowing that the first records of COVID-19 in Brazil date from the 8th epidemiological week of 2020. For this reason, the period analyzed includes epidemiological data from the weeks 8 to 53 of 2020 (16/02/2020 to 02/01/2021) and weeks 1 to 15 of 2021 (01/03/2021 to 17/04/2021).

The variables chosen were: age, gestational period (first, second, third trimester or postpartum), type and number of comorbidities (heart disease, kidney disease, neurological disease, hematological disease, liver disease, diabetes, asthma, obesity, chronic lung disease and immunosuppression), skin color (yellow, white, brown, black and indigenous), to better visualize the spatial distribution of deaths resulting from cases classified as COVID-19.

They were conducted using the free statistical software R (R Foundation for Statistical Computing Platform, version 4.0.3) and Statistical Package for Social Science (SPSS) version 29.0. SIVEP-Gripe notifications that evolved to death due to confirmation of COVID-19 were analyzed, a chi-square test was performed to determine the association between the analyzed variables and the classification of COVID-19 cases. When the observed frequency was less than 5 cases, Fisher’s test was performed. In addition, Odds Ratios (OR) and respective confidence intervals (95% IC) were calculated. In cases where the quantitative variables showed an approximate symmetrical behavior, the comparison of means was performed using the t-test. All inferential analyzes were considered significant at a 5% threshold.

The preservation of ethical aspects was ensured, in accordance with Resolution 510 of the National Health Council, of April 7, 2016, sole paragraph, which states that they will not be registered or evaluated by the Research Ethics Committee/National Commission system of Ethics in Research (CEP/CONEP), in item II, research that uses publicly accessible information, pursuant to Law No. 12.527, of November 18, 2011. It is noteworthy that the database used is publicly accessible, does not contain the names of the participants or any other possibility of individual identification of pregnant and postpartum women, in order to guarantee anonymity. As this is a research with a publicly accessible database, it was not necessary to refer it to the Research Ethics Committee.

## Results

A total of 19,333 cases of pregnant and postpartum women aged between 10 and 55 years diagnosed with SARS were identified, whether due to confirmed COVID-19 or unspecific causes. Of these, 1,279 died, these cases were classified into two groups according to the cause of death: deaths from COVID-19 (n= 1,026) and deaths from SARS of unspecific cause (n= 253). As of September 2020, there has been a steady decline in the proportion of deaths classified as nonspecific. As we obtained only two classification groups, the complement of the proportion represented by each point corresponds to deaths classified by COVID-19. In the first month analyzed (February 2020), all deaths (n=3) corresponded to nonspecific cases of SARS; this proportion decreased by approximately 10% in the last month analyzed (March 2021), meaning that the remaining 90% of deaths were cases of SARS related to COVID-19. Table 1 shows that the mean maternal age was higher in patients with confirmed death from COVID-19 than from unspecific causes (32.24 ± 7.49 years vs. 30.19 ± 9.09 years; p= <0.001). Regarding race, we noticed a higher proportion of black pregnant and puerperal women in the group with death from nonspecific causes than in the COVID-19 group, while ethnicities were present with similar percentages in both groups.

**Table 1:** Sociodemographic characteristics of pregnant and postpartum women with SARS due to COVID-19 or unspecific cause

Characteristics	Numbers	SARS caused by COVID-19	No specific by SARS	p-value
Age (years)				
Mean ± S.D.	-	32.24 ± 7.49	30.19 ± 9.09	0.0010
Age group (years)				<0.0001
<20	67	36(3.5)	31(12.3)	
20–34	737	593(57.8)	144(56.9)	
>35	475	397(38.7)	78(30.8)	
Color				0.0183
White	384	311(34.3)	73(34.3)	
Black	105	73(8.0)	32(15.0)	
Yellow	12	11(1.2)	1(0.5)	
Brown	609	505(55.7)	104(48.8)	
Indigenous	10	7(0.8)	3(1.4)	
Education				<0.0001
Residence area				0.0398
Rural	104	75(8.2)	29(12.6)	
Urban	1150	845(91.8)	201(87.4)	
Gestational moment				<0.0001
1st trimester	72	45(4.4)	27(10.7)	
2nd trimester	259	214(20.9)	45(17.8)	
3rd trimester	432	376(36.6)	56(22.1)	
Unknown	50	42(4.1)	8(3.2)	
Puerperium	466	349(34.0)	117(46.2)	

Source: Prepared by the authors with data extracted from the *Sistema de Informação para a Vigilância Epidemiológica da Gripe* (https://opendatasus.saude.gov.br/dataset/srag-2020). Brazil, 2020-2021.

In table 2, when comparing the type of symptoms in both groups, we observe symptoms more reported in patients who died of SARS caused by COVID-19, that is, fever (68.9% vs. 55.9%; p<0.0001), cough (79.4% vs. 57.8%; p <0.0001), fatigue (35.7% vs. 19.4%; p=0.0023), loss of smell (16.4 % vs. 5.2%; p= 0.0028) and loss of taste (15.2% vs. 5.2%; p=0.0062). In particular, the prevalence of loss of smell and taste among confirmed COVID-19 cases is more than three times higher than in cases of unspecific cause. The presence of nosocomial infection was also significantly different (3.2% vs. 7.1%; p= 0.0264), being higher in the group of deaths from nonspecific causes. Symptoms such as dyspnea, respiratory distress, lower oxygen saturation, and others did not show significant differences between groups. Among pregnant or postpartum women who died of SARS, those in the COVID-19 group had a higher ICU admission rate (77.6% vs. 69.2%; p=0.0104). The frequency of orotracheal intubation was similar between groups (66.6% vs. 60.0%; p=0.0746). Among patients admitted to the ICU, both the mean length of stay and the time elapsed between the onset of symptoms and the date of death were significantly longer for the COVID-19 group (13.61 ± 14.25 days vs. 7.70 ± 12.88 days, p<0.001 and 20.00 ± 15.04 days vs. 11.53 ± 13.45 days, p<0.001, respectively). The maternal mortality ratio (number of maternal deaths/100,000 live births estimated based on the number of live births in 2019) due to COVID-19 and due to an unidentified cause is heterogeneous throughout Brazil. Maternal death rates from COVID-19 are higher in the states of Roraima and Amazonas (northern region), and the rate of death from unspecific causes is higher in Paraíba, Pernambuco, and Bahia (northeast region).

**Table 2:** Characteristics of comorbidities in pregnant and postpartum women with SARS due to COVID-19 or unspecific cause

Symptom	Numbers	SARS caused by COVID-19 (%)	No specific by SARS (%)	OR (95% IC)	p-value
Fever	1091	611/887 (68.9)	114/204 (55.9)	1.75 (1.28–2.38)	<0.0001
Cough	1129	729/918 (79.4)	122/211 (57.8)	2.81 (2.05–3.86)	<0.0001
Throat pain	929	193/750 (25.7)	33/179 (18.4)	1.53 (1.02–2.31)	0.0515
Dyspnea	1164	807/939 (85.9)	191/225 (84.9)	1.09 (0.72–1.64)	0.7643
Respiratory discomfort	1112	690/893 (77.3)	173/219 (79.0)	0.90 (0.63–1.30)	0.6461
O2 saturation <95%	1101	695/890 (78.1)	153/211 (72.5)	1.35 (0.96–1.90)	0.1009
Diarrhea	919	110/741 (14.8)	20/178 (11.2)	1.38 (0.83–2.29)	0.2624
Vomiting	916	91/733 (12.4)	33/183 (18.0)	0.64 (0.42–1.00)	0.062
Abdominal pain	652	59/554 (10.6)	10/98 (10.2)	1.05 (0.52–2.13)	0.9999
Fatigue	670	204/572 (35.7)	19/98 (19.4)	2.30 (1.36–3.91)	0.0023
Loss of smell	658	92/562 (16.4)	5/96 (5.2)	3.56 (1.41–11.52)	0.0028
Loss of taste	660	86/564 (15.2)	5/96 (5.2)	3.27 (1.30-10.61)	0.0062

Source: Prepared by the authors with data extracted from the *Sistema de Informação para a Vigilância Epidemiológica da Gripe* (https://opendatasus.saude.gov.br/dataset/srag-2020)*. Brazil*, 2020-2021.

Table 3 shows that in the analyzed groups, the occurrence of obesity was different (24.2% vs. 10.9%; p=0.0017), being more prevalent in deaths due to SARS. The occurrence of chronic lung disease between groups was also different (2.0% vs. 7.0%; p= 0.0071) the chance of having a case of chronic lung disease among deaths from SARS caused by COVID-19 was approximately one-quarter that among deaths from non-SARS-specific causes (OR 0.27; 95% IC: 0.11–0.67).

**Table 3:** Characteristics of symptoms presented by pregnant and postpartum women with SARS due to COVID-19 or unspecific cause

Comorbidity	Numbers	SARS caused by COVID-19 (%)	No specific by SARS (%)	OR (95% IC)	p-value
Heart disease	670	118/529 (22.3)	43/141 (30.5)	0.65 (0.43–1.00)	0.0559
Diabetes	670	117/534 (21.9)	24/136 (17.6)	1.31 (0.81–2.13)	0.3315
Obesity	662	129/534 (24.2)	14/128 (10.9)	2.59 (1.44–4.68)	0.0017
Asthma	643	43/513 (8.4)	8 /130 (6.2)	1.40 (0.64–3.05)	0.5101
Chronic hematological disease	636	13/508 (2.6)	6/128 (4.7)	0.53 (0.20–1.43)	0.3302
Chronic liver disease	631	7/502 (1.4)	3/129 (2.3)	0.59 (0.13–3.61)	0.4349
Chronic kidney disease	627	13/498 (2.6)	5/129 (3.9)	0.67 (0.22–2.43)	0.3904
Neurological disease	630	6/502 (1.2)	5/128 (3.9)	0.30 (0.074–1.26)	0.5196
Chronic lung disease	631	10/503 (2.0)	9/128 (7.0)	0.27 (0.11–0.67)	0.0071
Immunodepression	635	22/506 (4.3)	7/129 (5.4)	0.79 (0.33–1.90)	0.7737

Source: Prepared by the authors with data extracted from the *Sistema de Informação para a Vigilância Epidemiológica da Gripe* (https://opendatasus.saude.gov.br/dataset/srag-2020)*. Brazil*, 2020-2021.

## Discussion

From February 2020 to April 2021, there were 1,279 maternal deaths, of which 253 (19.8%) had no specific cause. The number of cases of unspecified causes decreased during the evaluation period. In addition, there is an uneven distribution of unspecified cases among Brazilian states, which may indicate less testing or greater difficulty in accessing healthcare in certain regions. It highlights the sociodemographic and clinical aspects of limited access to health, from diagnosis to treatment, faced by pregnant women in Brazil. At the beginning of the pandemic, the lack of adequate testing and the consequent overcrowding of health services may have had an impact on mortality from nonspecific causes. As the pandemic progresses and tests become more available, the number of deaths from non-specific SARS has decreased, but even in the last months analyzed (February and March 2021), these cases are still high, at 7.7%. This drop is due to the fact that pregnant women have been tested more frequently since they were classified in the risk group.

The incidence in women under 20 years of age was higher in the group with no specific cause than in the COVID-19 group, as well as in the proportion of black women. Previous studies associated younger maternal age, non-white, unemployed, less educated, living in northern areas.^(31)^ These disadvantages are characteristics of the inequalities associated with reduced access to health systems.^(32)^

Mortality from COVID-19 was higher among black Brazilian women.^(32)^ However, special attention should be given to the frequent inclusion of black women in the group of non-specific SARS pathogens. Black women tend to be illiterate and have limited access to health services.^(33-35)^ This suggests that this group has less awareness of symptoms and even less access to diagnostic tests than women and white women. In general, our findings that the group with nonspecific causes had a higher proportion of young and black women suggest that this group is made up of women with difficult access to health systems, which may explain the lack of an unexplained cause.^(32-35)^ In addition, unemployment, lower education, lower income, living in the northern region, rural living conditions and not having a health plan are characteristics of inequality associated with lower access to the health system.^(32)^ Taken together, our findings of higher frequencies of younger, black women and unspecific causes lead us to infer that this group is composed of women with difficult access to the health system and this may explain the lack of etiological diagnosis of SARS.

It is observed that obesity was 2.59 times more frequent in the group of deaths from COVID-19. One explanation is that this condition was one of the first to be identified as a risk factor for worse evolution of COVID-19, which may have led to prioritization of the obese population in the diagnosis of COVID-19.^(33-35)^ The higher frequency of lung disease in the death from non-specific SARS cause group may have occurred because some SARS symptoms may have been misinterpreted as a clinical exacerbation of the underlying disease and therefore the COVID-19 diagnostic test could have been more neglected in these cases.

The number of comorbidities constitutes a risk factor when considering the need for hospitalization in the ICU regarding the evolution to death. Clinical comorbidities were also identified in a prospective cohort study conducted in Turkey, they were present in 10 cases (34.5%), with obesity being the main condition (50%), followed by hypothyroidism (40%), so that the authors concluded that individuals with comorbidities are more susceptible to COVID-19. Having fever, tachypnea and tachycardia were the most common abnormal vital signs during admission: 27.6%, 24.1% and 27.6%, respectively. The study points out that individuals with comorbidities are more susceptible to COVID-19, however, it is considered that at the time of the study, the authors’ knowledge was limited to pregnant women.^(36)^

In a study carried out in England, Northern Ireland and Scotland, among pregnant women hospitalized for SARS-CoV-2, one third had pre-existing comorbidities, the main ones being obesity, hypertension and diabetes.^(37)^ Research reinforces that pregnant women with diseases such as hypertension, diabetes mellitus and asthma are more susceptible to the virus and have a more severe course of the disease, leading to respiratory failure and the need for mechanical ventilation.^(36,37)^ An American study with 46 pregnant women with SARS-CoV-2 infection found that approximately two thirds of them were overweight (28.6%, n=12, 28.6%) or obese (n=15, 35.7%), with two women meeting the criteria for class III obesity (BMI≥40). Although most pregnant women were healthy, 12 (26.1%) had comorbidities, such as type 2 diabetes (n=3), asthma (n=4), hypothyroidism (n=2) and hypertension (n=2). Thus, most patients with severe disease were overweight or obese before pregnancy, and half had asthma and conditions associated with obesity, such as hypertension. Notably, one in seven pregnant patients was hospitalized for breathing problems and one in eight had severe COVID-19. The authors concluded that obesity is a particularly worrying risk factor, as the national prevalence of obesity is 39.7%.^(38)^

A prospective cohort study carried out using data from the United Kingdom Obstetric Surveillance System found that 75 (46%) of the 162 women were from minority ethnic groups and black. The study showed that the incidence of admission with confirmed SARS-CoV-2 infection during pregnancy seemed to vary according to the women’s ethnic group, age and body mass index, since more than half of the pregnant women hospitalized for SARS-CoV-2 were black or from other ethnic minority groups, 70% were overweight or obese, 40% were aged 35 years or older, and a third had pre-existing comorbidities, the main ones being obesity, hypertension, and diabetes.^(37)^

This study investigated a large number of maternal deaths using a national database of hospitalized patients with data collected since the onset of the COVID-19 pandemic in Brazil. As this is a national database study, there are some limitations, mainly related to the completeness and quality of the information added to the form fields, often missing data. In addition, we cannot infer causal relationships between the variables , nor address the quality of care provided to each group.

## Conclusion

The data presented here draw attention to the number of deaths from SARS, especially among sociodemographic profiles, precarious access to health, such as the black population. In addition, limitations in adequate access to health care are reinforced by even lower rates of ICU admissions among women who died from SARS of an unspecified cause. Thus, it is concluded that in Brazil, the estimated mortality rate is higher than that reported in other countries and the probability of death increases with length of stay. The risk of death increased among black and brown women, in the postpartum period and with the presence of comorbidities, mainly diabetes, cardiovascular diseases and obesity. In addition, pregnant and puerperal women with hypoxemia and requiring ventilator support were more likely to die. The high mortality of obstetric populations reinforces the need to be vaccinated against COVID-19, as safety and efficacy during the pregnancy-puerperal cycle are established and recommended by the Ministry of Health, as well as safe, ethical, transparent and continuous monitoring of cases. and deaths from COVID-19, even after the adoption of generalized vaccination for this population.
